# Notes and News

**Published:** 1894-11-24

**Authors:** 


### Notes and News.

Collected and Communicated.

The Liverpool Hospital Sunday Fund collection amounted to £6,264 this year.

A special meeting of Governors of the Chelsea Hospital for Women will be held on Wednesday, November 28th, at half-past three, for the purpose of receiving the resignation of the Board of Management, to elect a new Board, and to receive the suggestions of the medical staff.

Whilst small-pox has shown so considerable a decrease in London, the number of cases amounting only to forty-seven in October, with two deaths, a certain prevalence is shown in other places. At the beginning of November twenty-seven fresh cases were reported in Edinburgh, with three deaths, and a still larger number of patients were reported in Dublin. At the latter place, sixteen persons were attacked in one commercial establishment in one day.

One or two extraordinary statements were made at a meeting of inquiry held to consider the question of extending the City Hospital for Infectious Diseases at Liverpool. One gentleman present, in opposing the extension, said that the houses in a street opposite were nearly all unoccupied, and that in an adjacent street hardly a week passed without a case of scarlet fever arising. No one present refuted this statement or offered any explanation affecting it. We think it almost certain that some other reason is to be found for the presence of the scarlet fever than the neighbourhood of the hospital, unless there has been some gross carelessness in the management. A study of the spot-maps issued by the Metropolitan Asylums Board shows that there is no excessive prevalence of fever around their hospitals. At the same meeting Dr. Whitford cast a doubt on the proper supervision of the institution by stating that he had observed parcels thrown over the hospital wall into the street, and others returned the same way. This gentleman's sense of honour was so great that he preferred the possible imperilling of lives to usurping the office of detective, and so had refrained from reporting the facts earlier.

Mr. G. E. Adams, who has been at the General Hospital, Cheltenham, has been appointed secretary to the West Ham Hospital, and will enter upon his duties in the beginning of January next.

A very excellent system has found its way into the educational method of poor children in several continental countries. What voluntary means have effected in this country in a large but unsystematic manner, is being accomplished by some foreign Governments. In France a system of school colonies has been established, which system provides means for poor children of large towns to enjoy a holiday time in the country. This is managed by Government grants. Last year 3,500 children were domiciled in the country for about three weeks. The expenditure, which we imagine included travelling expenses, was, we learn, at the rate of 2s. 8d. per day per head. The scheme is an excellent one, and might be adopted in England to the advantage of the nation; but even in this expensive country the cost of the scheme should be considerably less than that quoted in France.

Let the cobbler stick to his last, says a homely old proverb, and some readers will add, "Let the parson keep to theology," after reading a report in last week's issue. The provincial clergyman who opposed the medical officer's views on the treatment and prevention of infectious diseases does not appear to hold any qualification to practice medicine. This, however, did not prevent his informing the Kidderminster Board on the so-called common-sense way of dealing with measles and typhoid. To this the doctor responded by pleading for humane consideration and skilled treatment of the sick in "the house." Surely this will be granted, and also some immunity be guaranteed to the responsible medical officer from such attacks as the recent one on the discretion of his administration. Dr. Stretton may be congratulated on his able championship of the cause of the friendless poor.

We have to announce with regret the death of Mr. Albert Napper, F.R.C.S., the founder of cottage hospitals. Albert Napper's heart was in his work. He had a singularly clear intellect, a wise judgment, and was in many respects an ideal practitioner. His wisdom was displayed in the rules which he drew up for the regulation of the first cottage hospital. These rules provided a remedy against hospital abuse; enabled a patient to pay for his treatment according to his means; secured justice to the medical profession, and the maximum of comfort and care to the sick in rural districts. Cranleigh Village Hospital, the first cottage hospital, was opened thirty-five years ago, and since then some five hundred cottage hospitals have been established in this country, a signal testimony to the value of Albert Napper's work. His modesty was so great that it is very difficult to do justice to the man. Still the cottage hospital movement is a memorial of which any man might be proud. Its success will cause Albert Napper's name to be preserved upon the roll of those who deserve well of their day and generation, as well as in the annals of the profession which he loved so well.

